# A tellurium-based small compound ameliorates tumor metastasis by downregulating heparanase expression

**DOI:** 10.7150/jca.96001

**Published:** 2024-08-13

**Authors:** Yuan-Hao Liu, Li-Hsien Wu, Wen-Jun Fan, Shih-Han Chiu, Pei-Hsuan Chen, Chia C. Wang, Che-Hsin Lee

**Affiliations:** 1 Division of Cardiovascular Surgery, Department of Surgery, Kaohsiung Armed Forces General Hospital, Kaohsiung 80284, Taiwan.; 2 Department of Biological Sciences, National Sun Yat-sen University, Kaohsiung 80424, Taiwan.; 3 erosol Science Research Center, National Sun Yat-sen University, Kaohsiung 80424, Taiwan.; 4 Department of Chemistry, National Sun Yat-sen University, Kaohsiung 80424, Taiwan.; 5 College of Semiconductor and Advanced Technology Research, National Sun Yat-sen University, Kaohsiung 80424, Taiwan.; 6 Department of Medical Laboratory Science and Biotechnology, Kaohsiung Medical University, Kaohsiung 80708, Taiwan.; 7 Department of Medical Research, China Medical University Hospital, China Medical University, Taichung 404, Taiwan.

**Keywords:** tellurium, AS101, metastasis, heparanase, protein kinase-B, cell migration

## Abstract

Tellurium is a rare element, and ammonium trichloro (dioxoethylene-o,o') tellurate (AS101) is the most bioactive molecule among several synthetic tellurium compounds. AS101 was found to be immunomodulatory and can modulate types of cytokines. However, the effect of AS101 on tumor metastasis remains unclear. Heparanase, an endo-glucuronidase, cleaves heparin sulfate side chains of proteoglycans on the cell surface, further leading to the degradation of the extracellular matrix. Heparanase also releases angiogenic factors in the extracellular matrix, is overexpressed in tumor cells, and promotes tumor metastasis and angiogenesis. In this study, we investigated the effect of AS101 in 4T1 and CT26 cells, especially heparanase. Heparanase expression was downregulated in 4T1 and CT26 cells after treatment with AS101 *in vitro*. The protein level involved in the protein kinase-B/mammalian target of rapamycin (AKT/mTOR) signaling pathway also declined. Cell migration assays revealed the inhibitory effect of AS101 on migration. The results of this study indicate that AS101 inhibits tumor migration by downregulating heparanase through the AKT/mTOR signaling pathway and has positive effects *in vivo*.

## Introduction

Tellurium is a chalcogen metalloid element with several chemical properties similar to selenium, an essential trace element in humans, and is widely used in clinical trials. However, due to its rare contact with humans, there have been relatively few studies related to the biological effects of tellurium. Despite tellurium's limited role in biological systems, some tellurium compounds, including tellurates and organic tellurides, have been reported to have potential applications in medical treatments, providing candidates for novel drugs 1. Among these organic tellurium compounds, ammonium trichloro (dioxyethylene-O,O) tellurate (AS101) is probably the most commonly used organotellurium compound in medicine according to its biological activity 2. AS101 is a small compound that plays roles not only in bactericidal activity 3, 4 but also as an immunomodulator. Researchers have revealed that AS101, which has the unique oxidation state of Te(IV), is capable of inhibiting cytokines (such as interleukin (IL)-7, granulocyte macrophage-colony stimulating factor, and interferon-γ) and integrin of T_H_1 and T_H_17 cells to relieve autoimmune disease 5. Owing to its ability to suppress tumor progression, AS101 has been applied in adjuvant therapy as well. Mice were administered prolonged treatment of AS101 generally prior to high doses of DNA-damaging agents, increasing hematopoietic stem cell proliferation and thus contributing synergistic effects with antitumor drugs 6. Furthermore, several studies offer evidence of the antitumor effects of AS101 7. For example, research has shown that tumor cells become more sensitive to chemotherapy after treatment with AS101, which downregulates IL-10, thereby decreasing cell proliferation 8. Additionally, AS101 induces apoptosis and growth arrest by reducing protein kinase-B (AKT) phosphorylation 9. Recent studies have suggested that AS101 is involved in tumor growth, but few mention the mechanism related to metastasis, which is regulated by the AKT pathway as well. Previous reports have demonstrated that tumor metastasis can be inhibited by regulating the AKT pathway, leading to a reduction in heparanase 10. Heparanase, a biological macromolecule, belongs to the endo-β-D-glucuronidase family, with a precursor at 65 kDa and an active heterodimeric state. Accounting for remodeling the extracellular matrix (ECM) at the cell surface, heparan sulfate (HS) offers binding sites for numerous growth factors or cytokines, sequentially changing the properties of heparan sulfate proteoglycans (HSPG), which are the presence of long chains of heparan sulfate polysaccharides 11. When degraded by heparanase, HS releases these bound molecules, which can stimulate cell motility and contribute to tumor cell migration. Therefore, it has been proven that the enhancement of heparanase improves tumor progression and metastasis, and it has thus become a pivotal target for novel cancer therapy 12, 13. Herein, we demonstrated that AS101 downregulates heparanase through the AKT signaling pathway in murine breast cancer and colon carcinoma models (widely used for their highly metastatic properties), and it exhibits significant inhibition of tumor metastasis.

## Materials and Methods

### Cell culture

CT26 (adenocarcinoma of mouse colon) and 4T1 (carcinoma of mouse mammary gland) cells were cultivated in 7 to 10 mL Dulbecco's modified Eagle's medium with 1% penicillin‒streptomycin and 10% fetal bovine serum and placed in 10 cm dishes. The culture dishes were maintained in an incubator at 37 °C and 5% CO_2_.

### Cell viability

Cells were placed in 96-well plates (3 × 10^3^ cells/200 μL per well) and treated with different AS101 concentrations (0, 2, 4, 8 μM) for 24 hours. Afterward, cell viability was measured by WST-1 assay (Roche, West Sussex, UK).

### Gene transfection

Cells were cultured in 6-well plates at 5 × 10^5^ overnight (37 °C with 5% CO_2_), transfected with 5 μg myr-AKT plasmid (constitutively active AKT, which was kindly provided by Dr. Chiau-Yuang Tsai of the Department of Molecular Immunology, Osaka University, Japan) in Lipofectamine 2000 (Thermo Fisher Scientific, Waltham, MA, USA), and then incubated overnight at 37 °C and 5% CO_2_. Afterward, the cells were treated with AS101 for 16 hours, and the solution was used for Western blotting.

### Protein extraction and quantitative analysis

Treated CT26 and 4T1 cells were cultured in 6-well plates overnight and then washed with PBS. Cells were subsequently lysed by adding 100 μL of lysis buffer (PBS with a 100 × dilution of protease inhibitor and 0.5% NP40 in each well). The resulting suspension was placed on ice for 30 minutes, followed by centrifugation at 12,000 rpm for 10 minutes at 4 °C. Protein content was analyzed using the Pierce^TM^ BCA Protein Assay (Pierce Biotechnology, Rockford, IL, USA).

### Western blot analysis

Proteins (40-80 μg/mL) were fractionated by SDS‒PAGE and then transferred onto membranes (Pall Life Science, Glen Cove, NY, USA). The membranes were probed with antibodies against heparanase (Santa Cruz Biotechnology, Santa Cruz, CA, USA), phosphorylated AKT (Santa Cruz Biotechnology), AKT (Santa Cruz Biotechnology), phosphorylated mTOR (Cell Signaling, Danvers, MA, USA), mTOR (Cell Signaling), or β-actin (Sigma Aldrich, St. Louis, MO, USA). The secondary antibodies used were anti-rabbit IgG or anti-mouse IgG (Jackson, West Grove, PA, USA). The enhanced chemiluminescence system was employed to visualize the signals, which were subsequently quantified using ImageJ software.

### Wound healing assay and Transwell assay

Cells were cultured in 12-well culture inserts (10^4^ cells/200 μL per well) placed in 12-well plates overnight. Every side of the culture insert was treated with 1 mL of AS101 at a concentration of 8 μM for 24 hours. Wound healing was compared between 0 and 8 μM AS101 at 0, 12 and 24 hours by photomicrographs. Cell migration was assessed according to the manufacturer's instructions for Transwell cultures (Thermo Fisher Scientific). Culture inserts were seeded with 2 × 10^5^ tumor cells in 200 μL of cell solution and placed in 24-well plates with 500 μL of medium added to each well. After that, half of the wells were treated with 8 μM AS101 for 24 hours, and then the cells in the culture inserts were fixed with formaldehyde for 3 mins. Penetrated cells were stained with 4',6-diamidino-2-phenylindole (DAPI). Finally, the cells were observed under a fluorescence microscope.

### Animal study

We purchased BALB/cByJNarl mice of approximately 5 weeks of age from the National Laboratory Animal Center of Taiwan. Animal studies were conducted with the approval of The Laboratory Animal Care and Use Committee of National Sun Yat-sen University (permit number: 10829). Tumor cells (1 × 10^5^) in 100 μL medium were treated with AS101 (8 μM) for 24 hours. Subsequently, these cells were inoculated into some mice over 6 weeks of age via the tail vein. Mice injected with CT26 and 4T1 cell lines were sacrificed on Day 15 or Day 20, respectively, after inoculation. Lung weights were recorded and used as samples for Western blot analysis as follows. The remaining mice were observed every day, and survival and mortality were recorded.

### Statistical analysis

The images were analyzed using ImageJ software, and the experimental data were processed using SigmaPlot statistical software and are presented as the mean ± standard deviation (SD). The p values for differences among means were determined using Student's t test, with statistical significance set at p < 0.05.

## Results

### Cell Viability of 4T1 and CT26 cells through AS101 treatment

**Figure 1:**
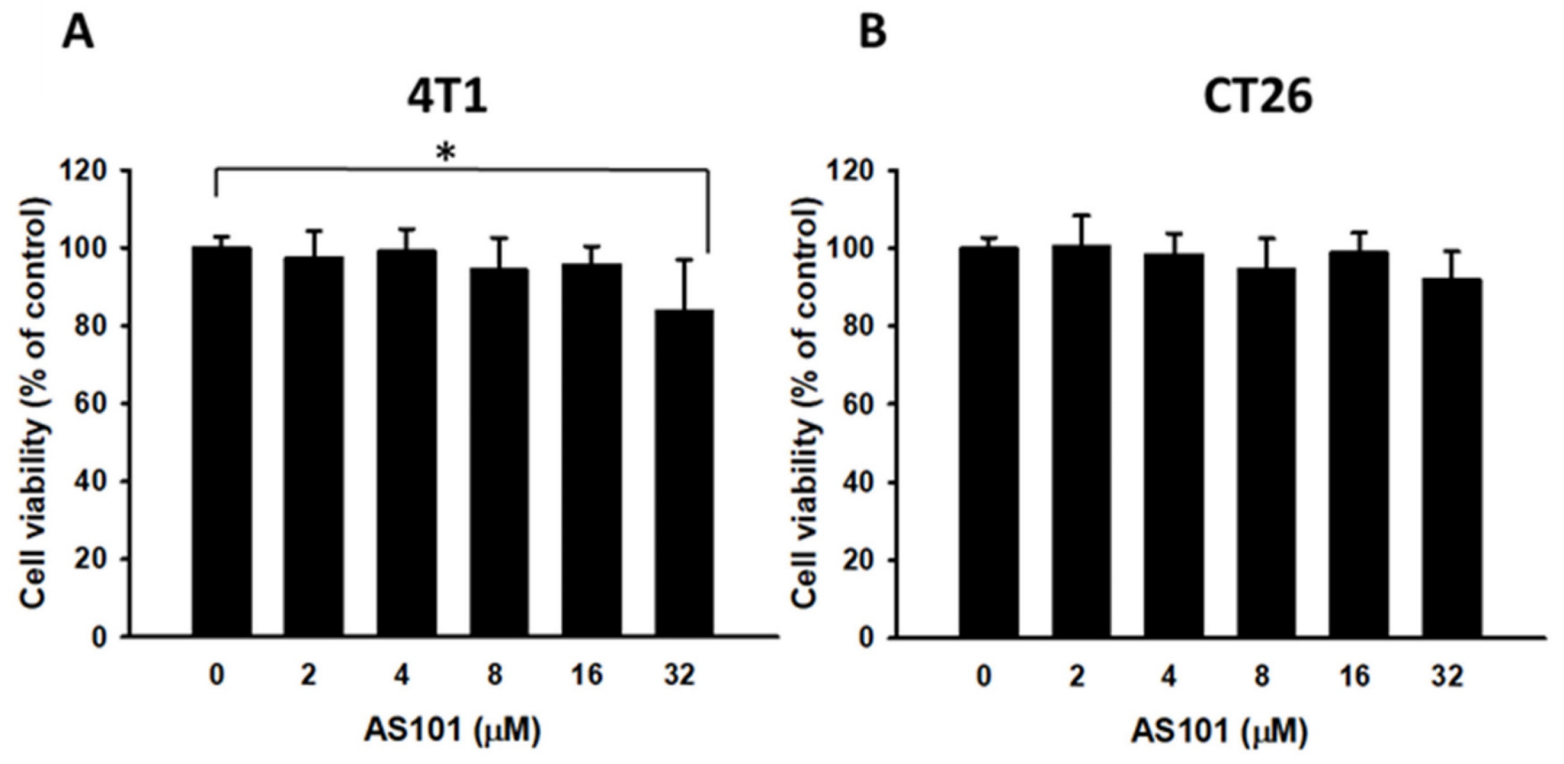
Cell viability of 4T1 and CT26 cells after treatment with AS101 for 24 hours. The viability of 4T1 (A) and CT26 (B) cells treated with various concentrations for 24 hours was measured by the proliferation reagent WST-1 (mean ± SD, n=4).

To determine the optimal concentration of AS101 that can yield the most significant results, cells were exposed to various AS101 concentrations ranging from 0 to 32 μM. The cell survival of 4T1 and CT26 cells treated with AS101 was measured using cell proliferation reagent (WST-1), as shown in Fig. 1. According to the statistics, there were no significant differences in viability among the concentrations (0-8 μM). At 32 μM, AS101 decreased the 4T1 cell viability. Therefore, subsequent experiments were conducted using the concentrations at 0, 2, 4, and 8 μM.

### AS101 reduced heparanase expression through the protein kinase-B/mammalian target of rapamycin (AKT/mTOR) signaling pathway

**Figure 2:**
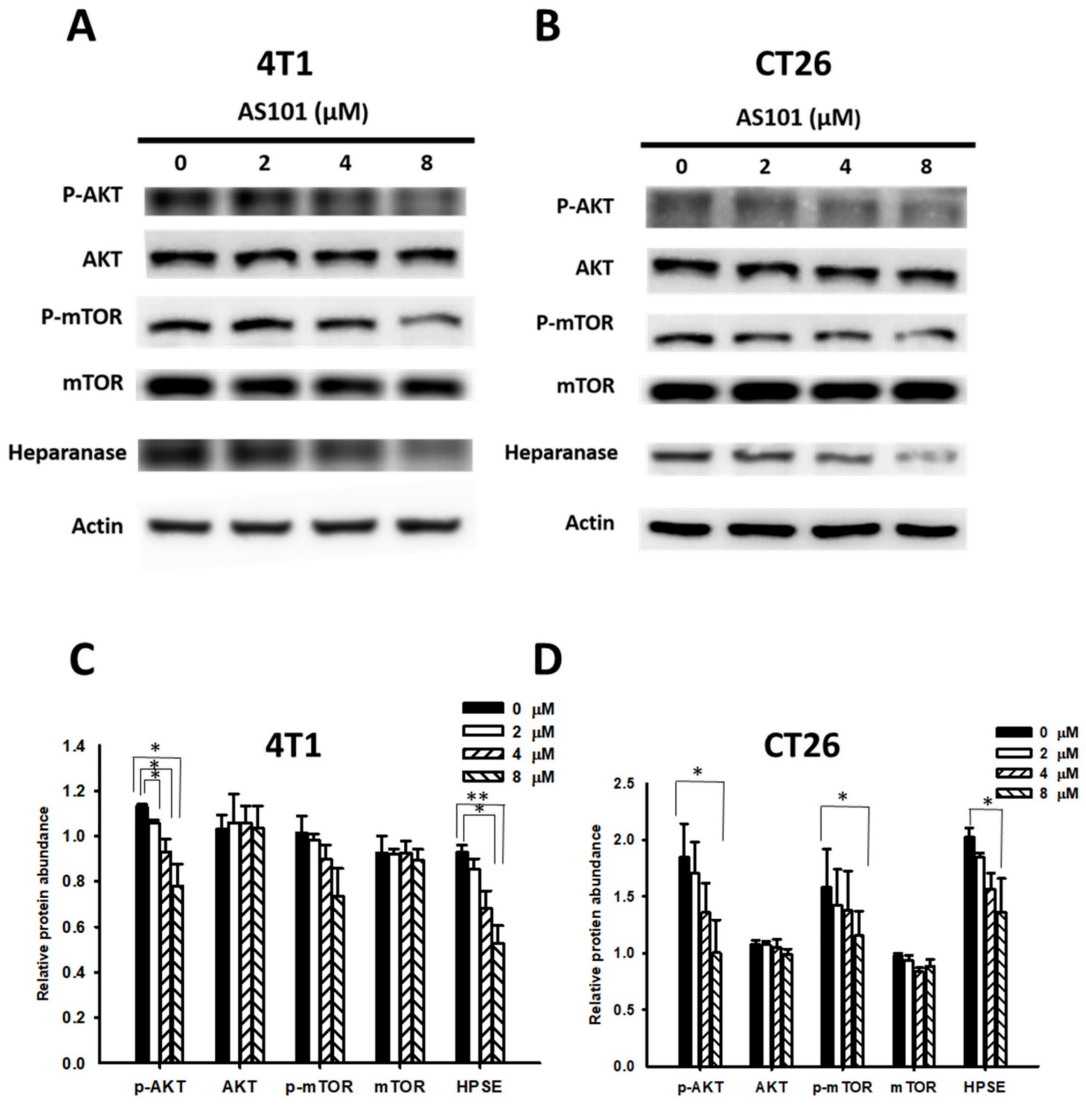
AS101 reduces the protein levels of heparanase and the AKT/mTOR pathway. 4T1 (A) and CT26 (B) cells were incubated with AS101 for 24 hours at different concentrations (0-8 μM). The protein levels of heparanase, AKT and mTOR were measured by Western blotting. Quantification histograms of 4T1 (C) and (D) CT26 are presented beneath each Western blotting plot. Data are expressed as the mean ± SD of three repeated determinations.

As mentioned previously, heparanase promotes tumor progression via the PI3K/AKT/mTOR signaling pathway, which is specifically correlated with the phosphorylation of AKT and mTOR. Furthermore, AS101 can inhibit the same pathway. Hence, we hypothesized that AS101 could influence the AKT/mTOR pathway, subsequently regulating heparanase levels, which might accelerate metastasis when increased. As shown in Fig. 2, we assessed the heparanase content and the expression of the AKT/mTOR pathway in CT26 and 4T1 cells treated with AS101 for 24 hours using Western blot analysis. It can be seen that the heparanase content decreased significantly as the concentration of AS101 increased, particularly at 8 μM. In addition, phospho-AKT and phospho-mTOR decreased due to the increase in AS101.

### AS101 inhibits the migration of tumor cells

**Figure 3:**
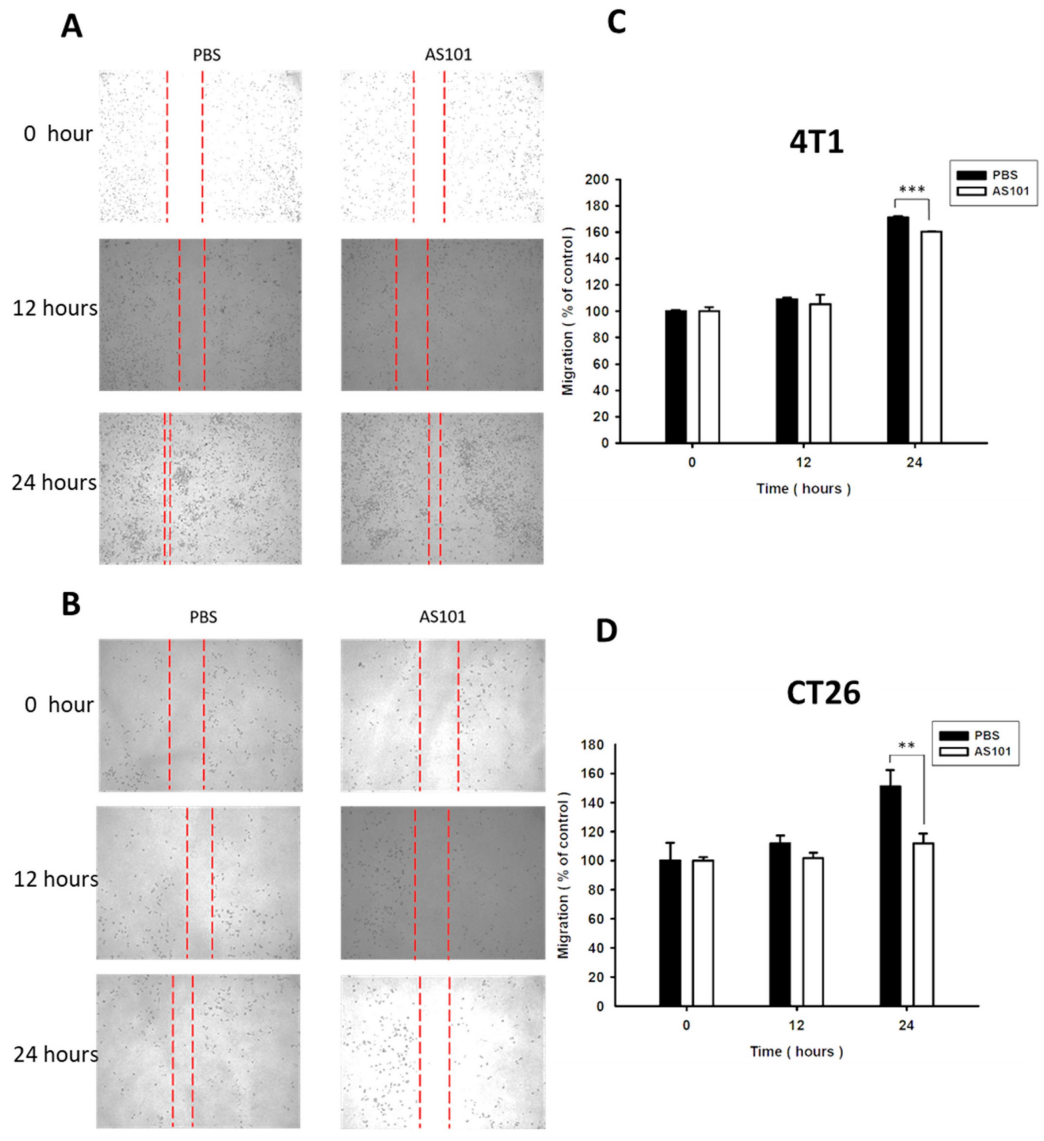
Condition of cell migration after treatment with AS101 detected by wound healing assay. 4T1 (A) and CT26 (B) cells (3×10^4^ cells per well) were placed into 2-well culture inserts inside 12-well plates and treated with 8 μM AS101 for 24 hours. The distances of both sides of the cells were measured at 0, 12, and 24 hours. The motility distances of different groups of B16F10 cells and CT26 cells were measured and are shown in (C, D). Data represent the mean ± SD, (n=3). *** P < 0.001; ** P< 0.01.

**Figure 4:**
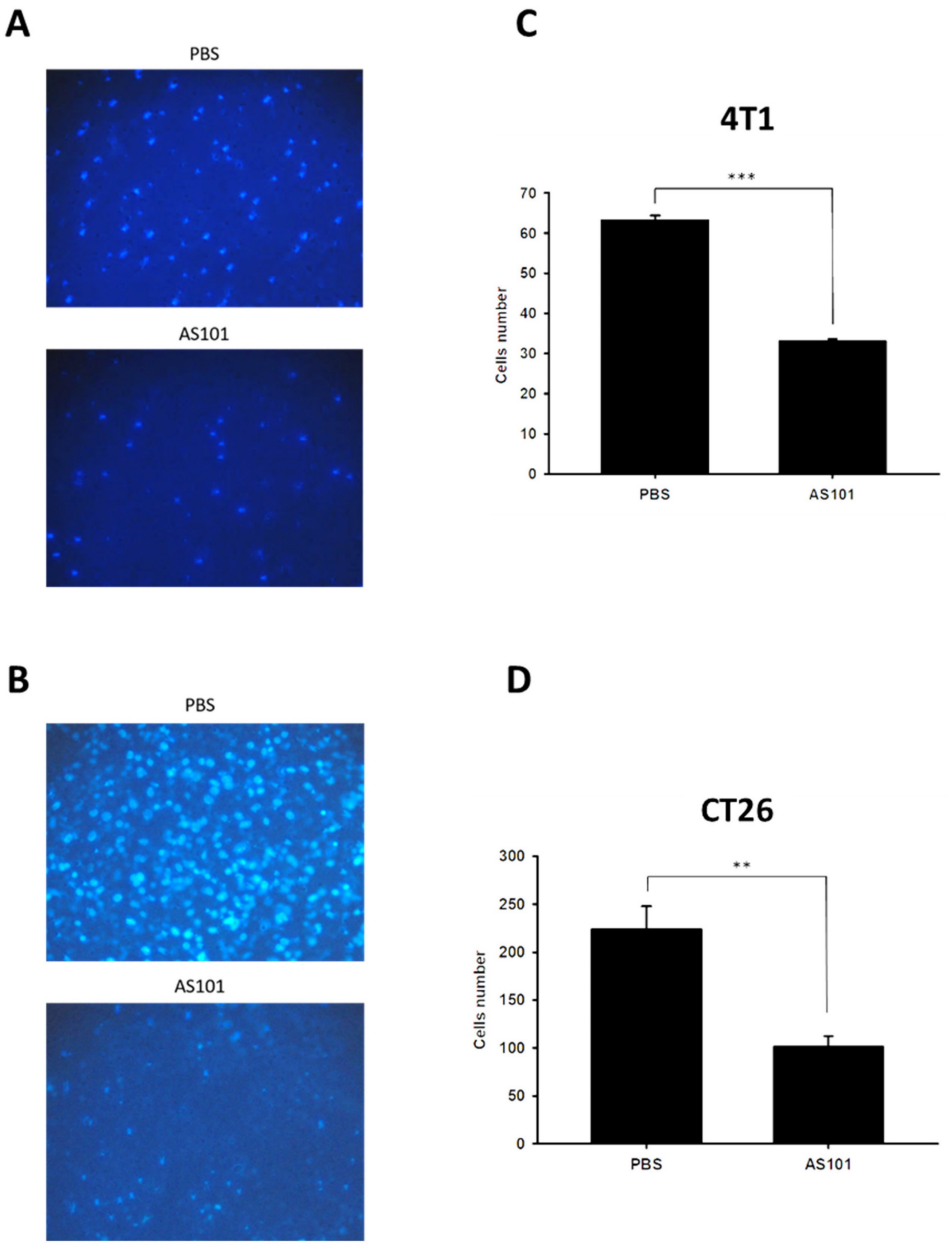
Condition of cell migration after treatment with AS101 detected by Transwell assay. 4T1 (A) and CT26 (B) cells (10^4^ cells per well) were placed into a culture insert with a permeable membrane inside a 24-well dish and incubated with 8 μM AS101 for 24 hours. The cells that passed through the permeable membrane were stained with DAPI and measured by fluorescence microscopy (200×). The motility of cells in different groups of 4T1 (C) and CT26 (D) cells was measured. Data represent the mean ± SD (n=3). *** P < 0.001; ** P< 0.01.

We found that heparanase is closely correlated with tumor metastasis and is overexpressed in several types of cancer. Heparanase is associated with the hallmarks of cancer. Existing research has shown that AS101 downregulates the expression of heparanase, leading to the inhibition of tumor cell metastasis. Accordingly, we used wound healing analysis to observe whether the migration ability of tumor cells decreases when treated with AS101. After treatment with AS101 (8 μM) for 0, 12, and 24 hours, we observed and analyzed the distance between the two clusters of cells. The results showed a nonsignificant difference between the 12-hour group and the control in both the 4T1 (Fig. 3A) and CT26 (Fig. 3B) cell lines. Additionally, after 24 hours, the ability of AS101 to inhibit cancer cell migration was evident, as it retarded the approach of two parts of cells. Moreover, the Transwell assay was employed to study the migration of tumor cells. Figure 4 clearly shows that the number of 4T1 cells (Fig. 4A) and CT26 cells (Fig. 4B) penetrating the membrane after 24 hours of AS101 treatment was lower than that of the non-treated cells. As a result, we concluded that AS101 reduces the migration of both types of tumor cells.

### AS101 decreased heparanase expression even under constitutive overactivation of the AKT/mTOR signaling pathway

Our earlier data demonstrated that AS101 reduced the level of heparanase by downregulating the AKT/mTOR pathway. To confirm whether the pathway influenced protein expression, we constitutively analyzed AKT gene expression. Using constitutive AKT expression, we activated the AKT pathway, which dramatically increased the content of heparanase. Subsequently, AS101 (8 μM) treatment was applied for 24 hours to assess heparanase expression and cell migration.

**Figure 5:**
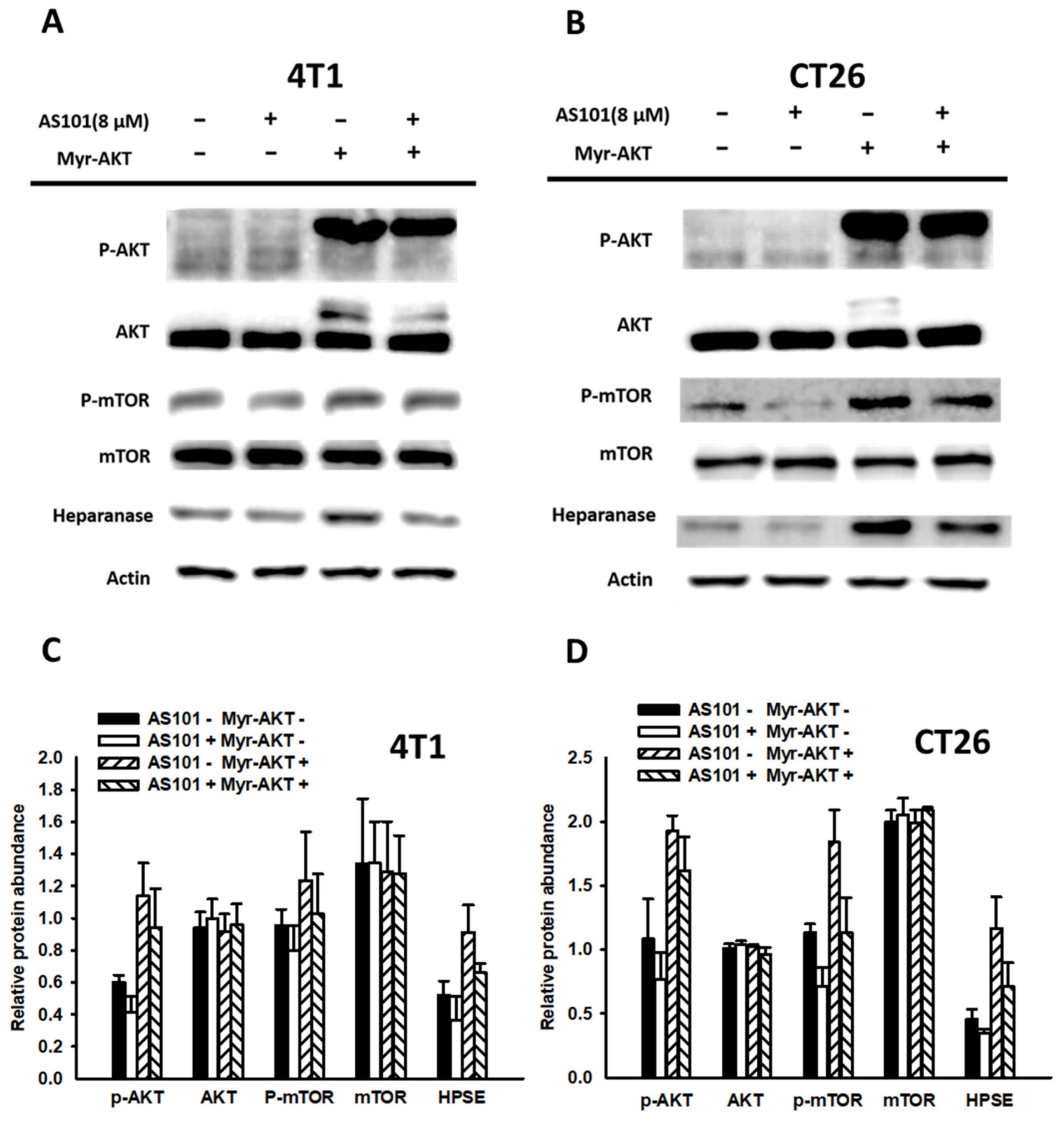
Correlation between heparanase and the AKT/mTOR signaling pathway. 4T1 (A) and CT26 (B) cells (5 ×10^4^ cells per well) were transfected with a myr-AKT plasmid, including constitutive AKT expression for 16 hours. Subsequently, the cells were treated with AS101 for an additional 24 hours. The protein levels of heparanase, AKT and mTOR were measured using Western blotting. Quantification histograms of 4T1 (C) and (D) CT26 are presented beneath each Western blotting plot. Data are expressed as the mean ± SD of three repeated determinations.

**Figure 6:**
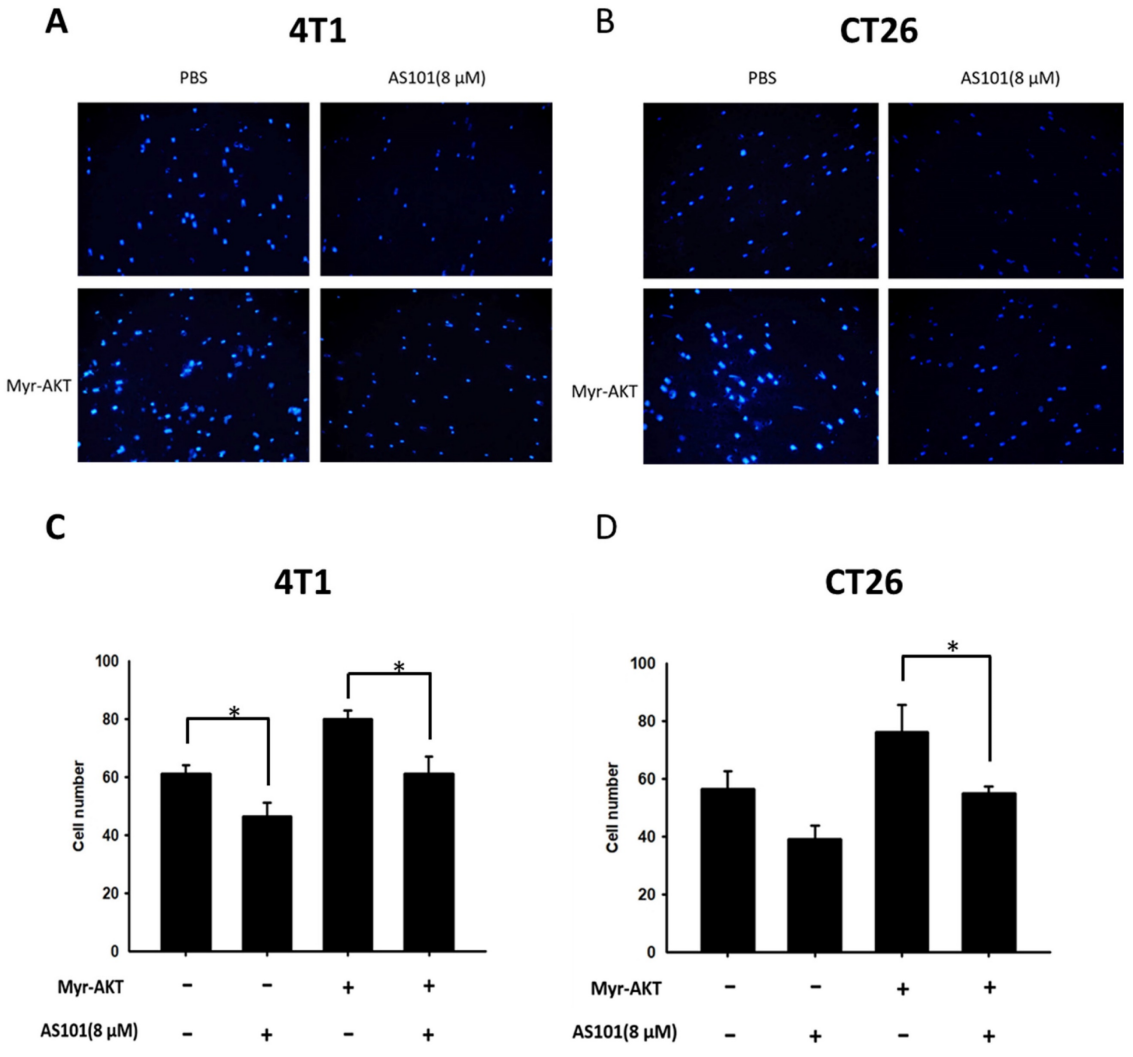
A Transwell assay showed that the AKT signaling pathways participated in the AS101-mediated inhibition of tumor cell migration. 4T1 (A) and CT26 (B) cells were transfected with active AKT plasmids. After 16 hours, the 4T1 and CT26 cells were placed on the upper layer of a transwell and then treated with AS101 (8 μM). After 24 hours, the bottom layer of cells was stained with 4',6-diamidino-2-phenylindole (DAPI) and counted under a fluorescence microscope in (C) 4T1 cells and (D) CT26 cells.

According to the results of the Western blot analysis (Fig. 5), the transfection group showed overactivation of the AKT/mTOR pathway. However, both the transfection and nontransfection groups exhibited significantly lower signal expression when treated with AS101 compared to those without AS101. Therefore, from the upregulation of signals and associated increase in heparanase, it is apparent that the AKT/mTOR pathway has a direct impact on heparanase and is regulated by the existence of AS101. In addition, a Transwell assay was conducted on those groups (Fig. 6). The results indicated that the number of transfected cells that penetrated the membrane was higher than that of the nontransfected group, but all the groups had a reduced number due to AS101.

### AS101 inhibited tumor metastasis and enhanced mouse survival

**Figure 7:**
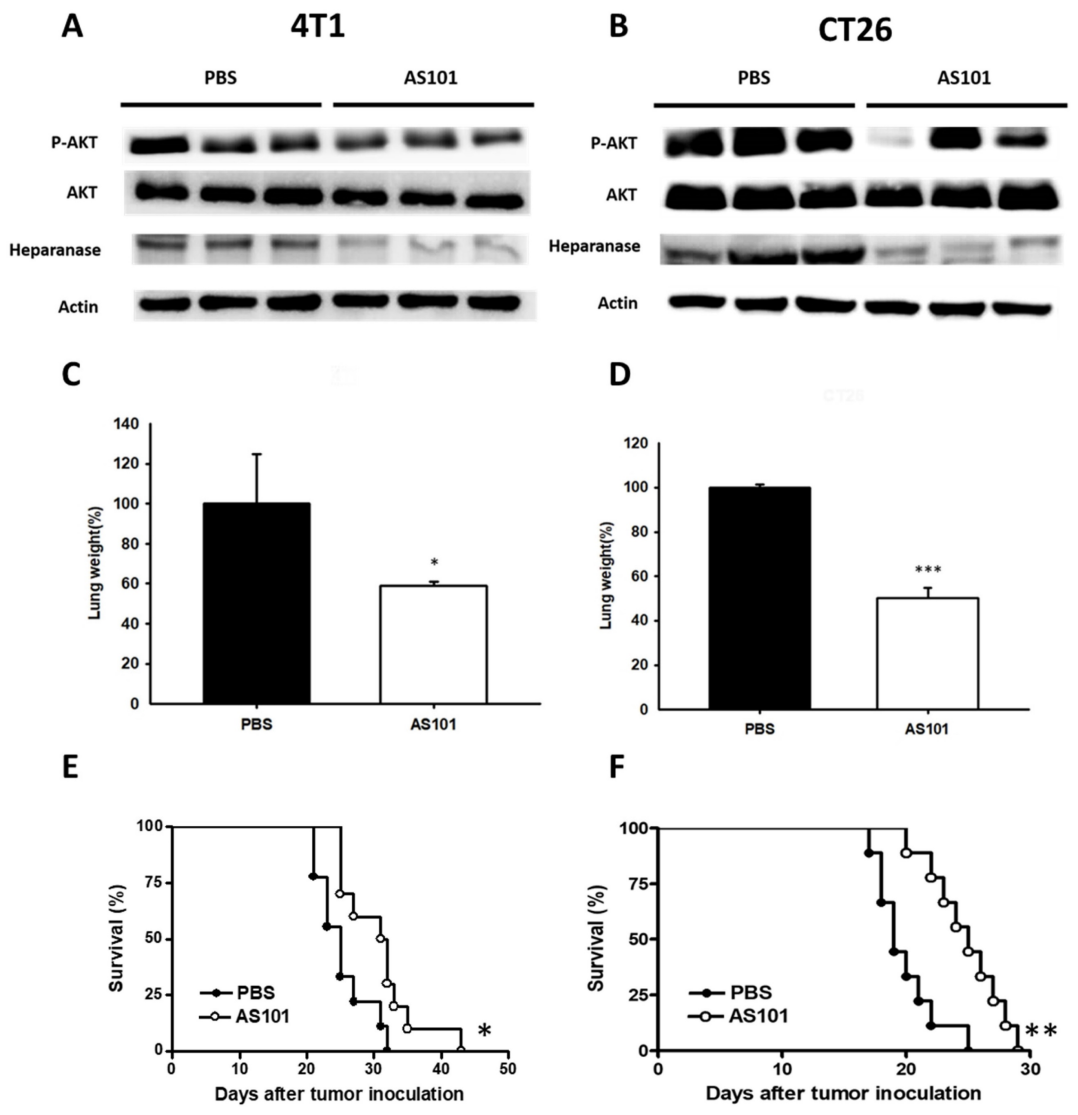
AS101 reduces tumor metastasis through downregulation of heparanase *in vivo.* 4T1 (A) and CT26 (B) cells were treated with 8 μM AS101 for 24 hours. Each mouse was injected with 10^5^ cells (treated and untreated) through intravenous injection. The mice were sacrificed at 21 days (4T1) and 15 days (CT26). The protein levels of heparanase, AKT, and mTOR in the lung were measured by Western blotting (n = 3). Inhibition of metastasis in tumors leads to lighter lung weight after treatment with AS101. The mice were sacrificed at 21 days (4T1, C) and 15 days (CT26, D). The lung weight was measured. (mean ± SD, n = 3) * P < 0.05, *** P < 0.001. The Kaplan‒Meier survival curves of mice bearing AS101-treated (E) 4T1 and (F) CT26 tumors are shown (n = 10, data are expressed as the mean ± SD. * P < 0.05, ** P < 0.01).

To ascertain the effect of AS101 *in vivo*, 4T1 and CT26 cells were treated with AS101 for 24 hours. Mice were sacrificed (mice injected with CT26 on Day 15 and 4T1 on Day 20), and their tissues were collected for Western blot protein analysis. The remaining mice were monitored for survival. However, the expression of phospho-AKT and heparanase was lower in AS10-treated 4T1 and CT26 tumor-bearing mice, as demonstrated by Western blotting (Fig. 7A, B). The results showed that the lung weight of mice injected with cells treated with AS101 was lower than that of mice without AS101 treatment (Fig. 7C, D). We inferred that the reduced number of tumor cells after AS101 treatment is capable of restraining the metastasis of tumors heading to the lungs. According to the image (Fig. 7A, B), the protein expression of lung cells followed the same trend as *in vitro* experiments. Hence, we have provided evidence that AS101 decreased the expression of heparanase, alleviating mouse lung by intercepting metastasis. From Fig. 7E and Fig. 7F depicting mouse survival, it can be seen that administration of AS101 successfully prolonged the lifespan of mice. Overall, we concluded that AS101 inhibits metastasis via downregulation of heparanase.

## Discussion

The metastasis of tumor cells is a crucial reason why single cancer therapy often proves ineffective, making cancer even more malignant and fatal, despite patients undergoing repeated treatments. Currently, employing multiple therapies has become a trend, offering potential relief from relapses and chemoresistance 14. In recent years, AS101 has contributed to effective trials combined with types of chemotherapy. Our study identified 8 μM as the highest concentration of AS101, and it manifested nontoxicity both *in vitro* and *in vitro*. However, in studies in which AS101 was administered to cancer patients, the dose with no apparent toxicity was 3 mg/m2 three times per week for 2 weeks 15. The dosage we display is far below that in clinical trials, evidencing that low doses can take part in the treatment course as well.

Recently, AS101 has been revealed to exhibit significant antibacterial activities, particularly against carbapenemase-producing *Escherichia coli* (CPEC). The prevalence and challenge of carbapenem-resistant *E. coli* have been escalating over the years, with carbapenemase-producing strains identified as particularly problematic due to their resistance mechanisms. The mechanistic investigations suggest that AS101 enters the bacterial cell and induces the generation of reactive oxygen species (ROS), which leads to DNA fragmentation 16.

On the one hand, in our study, we focused on the enzyme function of heparanase, which mainly leads to the migration, invasion, and angiogenesis of tumors through modifying HSPG and ECM 17, 18. On the other hand, heparanase displays non-enzymatic properties in numerous studies, influencing the tumor microenvironment. For instance, heparanase has been shown to activate specific signaling pathways, including the AKT/mTOR pathway. Activation of these pathways can enhance cell motility and contribute to the invasive properties of tumor cells. Furthermore, heparanase has other functions, including immunomodulatory effects 19. Heparanase can influence the behavior of immune cells and the secretion of cytokines, which are signaling molecules involved in immune responses. Intriguingly, antibodies have been reported to neutralize heparanase and thus restrain lymphoma tumor invasion and metastasis 20. Immunomodulatory functions of some agents can impact the tumor microenvironment and potentially influence cancer progression 21. Programmed Death-Ligand 1 (PD-L1), is a protein expressed on the surface of cells. In the context of cancer, PD-L1 can be found on the surface of tumor cells and tumor-infiltrating immune cells 22. It plays a crucial role in helping tumors evade the immune system. PD-L1 binds to the programmed death 1 (PD-1) receptor on T cells, an interaction that normally helps maintain immune tolerance and prevents autoimmunity 23. However, tumors exploit this pathway to protect themselves from immune attack. In this study, AS101 did not influence the protein expression of programmed cell death-ligand 1 (Figure S1). All things considered; it seems goal-oriented to assess the results related to reducing heparanase in clinical treatments.

## Conclusions

Our study indicated that downregulating the expression of heparanase with AS101 treatment significantly reduced the migration of mouse tumor cells *in vitro* and *in vivo*. By analysis of the pleiotropic activities of AS101, AS101 not only has antibacterial activity but also reduces tumor metastasis.

## Supplementary Material

Supplementary figure.
