# Supplementary material for: A tellurium-based small compound ameliorates tumor metastasis by downregulating heparanase expression
Source: J Cancer. 2024 Aug 13;15(16):5308–17. doi: 10.7150/jca.96001 (PMC11375552; doi:10.7150/jca.96001)
Supplement: Supplementary file 1 — Supplementary figure. [file jcav15p5308s1.pdf]

Figure S1

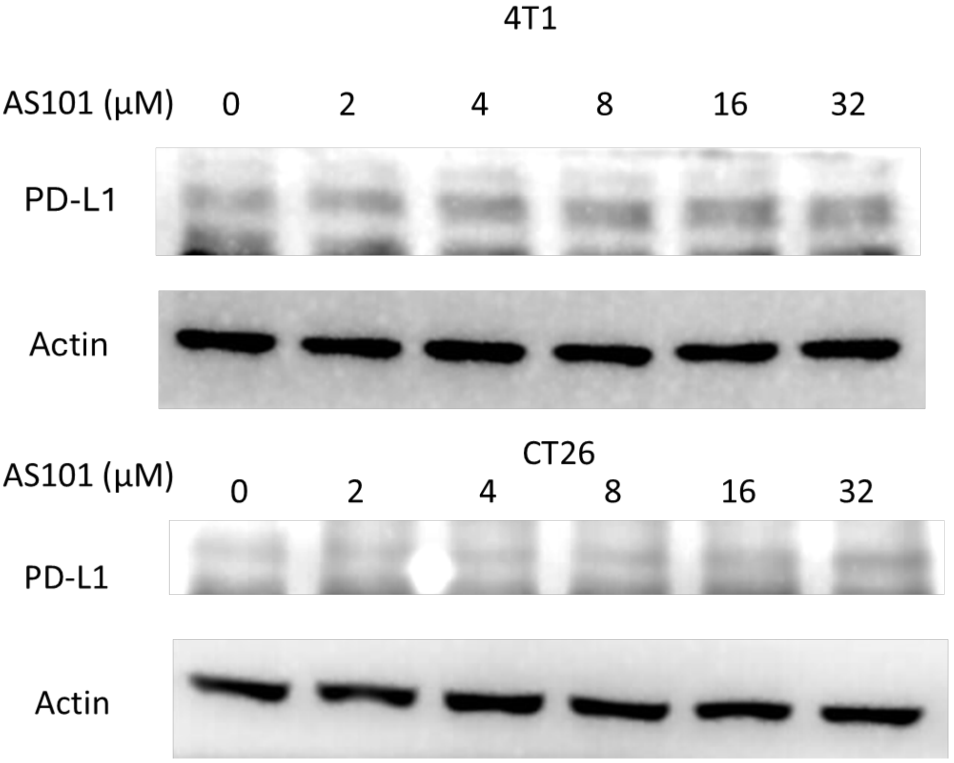

Figure S1. AS101 dose not reduce the protein levels of PD-L1. 4T1 (A) and CT26 (B) cells were incubated with AS101 for 24 hours at different concentrations (0-32 µM). The protein level of PD-L1 was measured by Western blotting.
